# Effect of Previous Cesarean Section on Ultrasound Doppler Studies of Placental Circulation and Pregnancy Outcome

**DOI:** 10.3390/jcm15124412

**Published:** 2026-06-07

**Authors:** Threebhorn Kamlungkuea, Woraluk Moradokkasem, Nareenun Chansriniyom, Chayada Tangshewinsirikul, Sommart Bumrungphuet, Theera Tongsong

**Affiliations:** 1 Department of Obstetrics and Gynecology, Faculty of Medicine Ramathibodi Hospital, Mahidol University, Bangkok 10400, Thailand; threebhorn.k@cmu.ac.th (T.K.);; 2 Department of Obstetrics and Gynecology, Faculty of Medicine, Chiang Mai University, Chiang Mai 50200, Thailand

**Keywords:** cesarean section, Doppler ultrasound study, uterine artery, pulsatility index (PI), resistance index (RI)

## Abstract

**Objectives:** To primarily determine the effect of a previous cesarean section on Doppler studies of the uterine artery, umbilical artery, and umbilical vein. **Patients and Methods:** This prospective study was conducted on singleton pregnancies between 18 and 22 weeks of gestation, including both women with and without a previous cesarean section. Doppler studies of the uterine artery, umbilical artery, and umbilical vein indices were performed during mid-trimester fetal ultrasound scans. Doppler indices and pregnancy outcomes were compared between the two groups. **Results:** A total of 351 pregnancies, including 74 women with a previous cesarean section and 277 women with an unscarred uterus, underwent Doppler studies. The uterine artery pulsatility index (PI) and resistance index (RI) were significantly higher in women with a previous cesarean section (*p* < 0.001). Moreover, both univariate and multivariate analyses demonstrated that a previous cesarean section was significantly associated with an increase in uterine artery PI, RI, and the rate of abnormal uterine artery PI, defined as values above the 95th percentile (*p* = 0.034). Other Doppler study results, as well as pregnancy outcomes, were comparable between the two groups. **Conclusions:** Pregnant women with a previous cesarean section have significantly higher uterine artery resistance, and a higher rate of abnormal uterine artery PI during mid-pregnancy. Therefore, a cesarean section may be detrimental to uterine arterial health. However, no significant adverse clinical outcomes were observed.

## 1. Introduction

Over the past few decades, cesarean section (CS) rates have been rising globally [1,2]. More than one in five women worldwide (21.1%), and over half of women in some regions, deliver by cesarean section. Previous studies have shown that cesarean delivery is associated with adverse maternal and neonatal outcomes in subsequent pregnancies. Maternal complications include impaired implantation, abnormal placentation, an increased risk of preeclampsia, and uterine rupture [3,4,5,6]. Adverse neonatal outcomes include unexplained stillbirth, perinatal death, and preterm birth [7,8,9,10].

The pathological changes in the uterus following cesarean section include distortion and widening of the lower uterine segment, residual suture material associated with foreign-body giant cell reaction, reduced myometrial thickness, and an increased degree of fibrosis [11]. These pathological alterations may contribute to impaired trophoblastic invasion, abnormal placentation, vascular malformation and fragility, increased uterine artery resistance, and reduced uterine blood flow. Consequently, these changes may impair uteroplacental function and increase the risk of adverse outcomes in subsequent pregnancies [12,13,14,15,16,17].

Normal fetal growth and favorable pregnancy outcomes depend largely on appropriate placental development and the integrity of two circulatory systems: the uteroplacental circulation on the maternal side and the fetoplacental circulation on the fetal side. Doppler ultrasound provides a non-invasive method for evaluating uteroplacental and fetoplacental blood flow through assessment of the uterine artery, which serves as a surrogate marker of the maternal placental circulation; the umbilical artery, which reflects the fetal placental circulation; and the umbilical vein, which represents blood flow from the placenta to the fetus. These Doppler parameters provide valuable predictive markers for placental insufficiency and adverse pregnancy outcomes [18,19,20,21,22]. Evidence from previous studies suggests that prior cesarean section may affect uterine artery Doppler indices, resulting in increased uterine artery resistance, reduced uterine artery blood flow, and elevated uterine artery pulsatility index (PI). However, only a limited number of studies have investigated the effect of previous CS on uterine artery resistance [15,16,17,23,24,25], and the reported findings remain inconsistent. Some studies have demonstrated an association between prior CS and increased uterine artery resistance [16,25], whereas others have reported no significant relationship [23,26]. Importantly, most previous studies did not perform multivariate analyses to adjust for potential confounding factors affecting uterine artery resistance [15,16,17,23,24]. A recent systematic review and meta-analysis incorporating six studies (both retrospective and prospective) suggested that previous CS may be associated with increased uterine artery resistance. However, the authors emphasized that the available evidence remains limited, and further well-designed studies are required to confirm these associations and clarify their clinical relevance [27]. Therefore, we conducted this prospective study to determine whether a previous cesarean section is associated with increased uterine artery resistance while strictly controlling for potential confounding factors. The primary objective of this study was to examine the impact of previous cesarean section on Doppler indices of the uterine artery, umbilical artery, and umbilical vein. The secondary objective was to evaluate its potential influence on pregnancy outcomes.

## 2. Materials and Methods

This prospective cohort study was conducted at Ramathibodi Hospital, Bangkok, Thailand, from February 2023 to January 2024. The study enrolled women with viable singleton pregnancies who underwent a mid-trimester fetal ultrasound examination between 18 and 22 weeks of gestation. Exclusion criteria included hypertensive disorders, autoimmune disease, cardiovascular disorders, renal insufficiency, pre-existing diabetes mellitus, a history of preeclampsia or pregnancy-induced hypertension, smoking, previous uterine surgery other than cesarean section, prior preterm birth, fetal structural or chromosomal abnormalities, refusal to participate or provide informed consent, and failure to obtain Doppler measurements. The study group consisted of pregnant women with a history of previous cesarean section, whereas the control group comprised pregnant women with an unscarred uterus. The primary outcomes were Doppler velocimetry indices of the three major placental circulatory vessels, including the uterine artery (PI and RI), umbilical artery (PI, RI, and Systolic/Diastolic [S/D] ratio), and umbilical vein (maximum velocity and flow). The indices were selected for investigation because of their practicality, reproducibility, and widespread use in both clinical practice and research, thereby facilitating comparisons across studies.

Gestational age was determined for all pregnancies using standard fetal sonographic biometry in accordance with the 2017 guidelines of the American College of Obstetricians and Gynecologists (ACOG) [28]. Mid-trimester fetal ultrasound examinations were performed in accordance with the 2022 practice guidelines of the International Society of Ultrasound in Obstetrics and Gynecology (ISUOG) [29]. Eligible participants without fetal anomalies or other exclusion criteria were subsequently enrolled in the study. The study was approved by the Institutional Review Board of the Faculty of Medicine, Ramathibodi Hospital, Mahidol University (Research ID: COA.MURA2023/44), and written informed consent was obtained from all participants. Maternal baseline characteristics were collected from antenatal records.

Doppler ultrasound assessments of the bilateral uterine arteries, umbilical artery, and umbilical vein were performed by two maternal–fetal medicine specialists certified by the Fetal Medicine Foundation. All examinations were conducted using GE Voluson^®^ E8 and E10 ultrasound systems (GE Healthcare Austria GmbH & Co OG, Zipf, Austria) equipped with 1–6 MHz convex transducers. Placental location was classified as anterior, posterior, lateral, or fundal when more than 50% of the placental mass was located within the respective region. Doppler indices of the bilateral uterine arteries and umbilical artery were measured transabdominally in accordance with the 2021 ISUOG Practice Guidelines for the Use of Doppler Velocimetry in Obstetrics [18]. The uterine artery was identified at the point where it crosses the external iliac artery. Measurements were obtained approximately 1 cm distal to the crossover point while maintaining an insonation angle of less than 30° until three consecutive similar waveforms were recorded. The uterine artery peak systolic velocity (PSV) was required to be at least 60 cm/s, as lower velocities may indicate assessment of an incorrect vessel or an inappropriate sampling position [30]. The same procedure was performed for the contralateral uterine artery. Umbilical artery Doppler indices were assessed at a free loop of the umbilical cord while maintaining an insonation angle of less than 30° until three consecutive similar waveforms were obtained. Doppler indices for both the uterine and umbilical arteries were automatically calculated using the software package integrated into the ultrasound system. The mean uterine artery PI and RI were calculated as the average values of the left and right uterine arteries. For assessment of umbilical venous flow (UVF), the diameter of the umbilical vein was measured at the straight intra-abdominal portion of the vessel before the first branching of the portal vein, using a transverse view of the upper fetal abdomen with image magnification exceeding 30%. The diameter was measured perpendicularly from the inner wall to the inner wall of the vessel lumen, and the mean of three measurements was recorded. Umbilical vein flow velocity was estimated by averaging two pulsed-wave Doppler measurements obtained during fetal quiescence for 2–4 s. Measurements were acquired with an insonation angle close to 0° or less than 15°, and the sample volume encompassed the full width of the umbilical vein. UVF was calculated using the following formula: The UVF was calculated as: 0.5 × time-averaged maximum velocity × π × (UV diameter/2)^2^ [26,31]. Participants were followed until delivery, and data regarding pregnancy outcomes and adverse pregnancy complications, including preeclampsia, fetal growth restriction, low birth weight, preterm birth, and oligohydramnios, were collected. Preeclampsia was diagnosed according to the 2019 guidelines on gestational hypertension and preeclampsia recommended by the ACOG [32]. Fetal growth restriction was defined according to the Delphi consensus criteria [33]. Additional outcomes included low birth weight (<2500 g), preterm birth (<37 weeks of gestation), and oligohydramnios, defined as an amniotic fluid index < 5 cm or a single deepest vertical pocket < 2 cm [34].

**Statistical analyses**: The sample size was calculated for the comparison of two independent means. The calculation was based on the assumptions that the mean uterine artery PI in the control group was 1.0 multiples of the median (MoM), the expected mean difference in PI between the two groups was 0.02 MoM, and the common standard deviation was 0.04. To achieve a statistical power of 90% at a 95% confidence level, the required sample size was estimated to be at least 57 participants in the study group and 228 participants in the control group, corresponding to an approximate study-to-control ratio of 1:3. Statistical analyses were conducted and reported utilizing Stata software version 16.0. Patient characteristics were summarized as mean ± standard deviation (SD) for continuous variables, while categorical variables were expressed in terms of proportions or percentages. The normality of the data distribution was evaluated using the Shapiro–Wilk test. Continuous variables were compared with Student’s t-test, and categorical variables were assessed with the chi-square test and Fisher’s exact test. Moreover, univariable and multivariable regression analyses were performed to investigate the associations between the variables. Covariates included in the multivariable models were selected based on their potential clinical relevance, and multicollinearity was assessed using variance inflation factor diagnostics. A *p*-value of less than 0.05 was considered statistically significant, with 95% confidence intervals (CIs) reported.

## 3. Results

**Figure 1:**
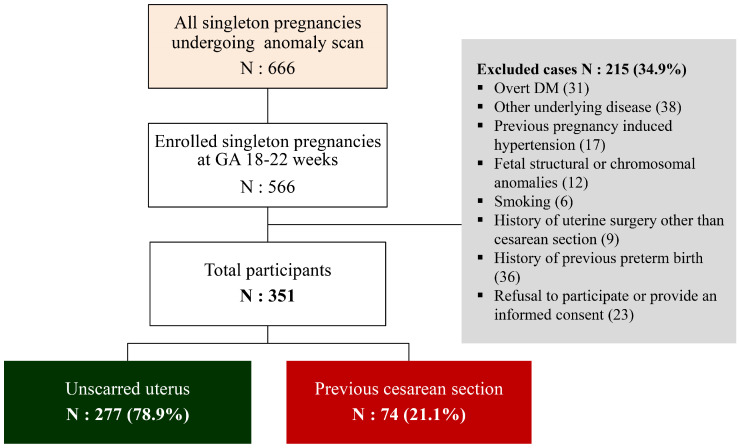
Flow diagram of patient recruitment and allocation.

A total of 351 women with singleton pregnancies were enrolled in this study. Of these, 74 women with a previous cesarean section were included in the study group, whereas 277 women with an unscarred uterus were recruited into the control group, as presented in Figure 1.

The baseline characteristics of both groups are compared in Table 1. The mean gestational age at enrollment and Doppler ultrasound assessment was 20.56 ± 0.07 weeks. Women with a previous cesarean section were significantly older than those with an unscarred uterus (33.6 ± 0.60 vs. 30.64 ± 0.30 years, *p* < 0.001). In addition, the study group had a significantly higher pre-pregnancy BMI and a greater proportion of women with advanced parity, previous abortion, and prior uterine curettage (*p* < 0.05). All previous cesarean sections involved a low-transverse uterine incision. The proportion of aspirin use was significantly higher in the control group than in the study group (13.71% vs. 2.7%, *p* < 0.001).

The uterine artery PI and RI were significantly higher in pregnant women with a previous cesarean section than in those with an unscarred uterus (1.11 ± 0.04 vs. 0.95 ± 0.02, *p* < 0.001, and 0.62 ± 0.01 vs. 0.56 ± 0.01, *p* < 0.01, respectively). In addition, the proportion of women with a uterine artery PI above the 95th percentile of the Thai normative reference values [35] was significantly greater among those with a previous cesarean section (13.51% vs. 6.14%, *p* < 0.001). However, no significant differences were observed in the Doppler indices of the umbilical artery or umbilical vein between the two groups, as presented in Table 2.

In both univariable and multivariable regression analyses, previous cesarean section was significantly associated with increased uterine artery PI and RI. The analyses demonstrated that each previous cesarean section was associated with an increase of 0.19 in the mean uterine artery PI (*p* < 0.001, 95% CI 0.104–0.277) and an increase of 0.058 in the mean uterine artery RI (*p* < 0.001, 95% CI 0.033–0.084). Furthermore, previous cesarean section was significantly associated with uterine artery PI above the 95th percentile in both univariable and multivariable logistic regression analyses (*p* = 0.029; 95% CI 1.04–5.46 and 1.09–10.10, respectively), as presented in Table 3.

Pregnancy outcomes are presented in Table 4. No significant differences were observed between the previous cesarean section and unscarred uterus groups with respect to fetal birth weight, placental weight, or adverse pregnancy outcomes, including pre-eclampsia, fetal growth restriction, low birth weight, preterm birth, and oligohydramnios.

## 4. Discussion

The main insight gained from this study is that cesarean section has a detrimental effect on uterine circulation, as indicated by a significant increase in resistance to uterine blood flow, reflected by elevated uterine artery PI, uterine artery RI, and a higher rate of abnormal uterine artery Doppler findings. This study provides evidence of subtle changes in uterine vessels after cesarean section, which is likely linked to underlying causes that explain the increased risk of various adverse obstetric outcomes. The effect of cesarean section on fetal circulation, if present, appears to be less pronounced than its effect on maternal circulation.

The main finding is an increase in uterine artery resistance as a consequence of a prior cesarean section, and this increase may be responsible for adverse outcomes in subsequent pregnancies. It is likely that the surgical procedure of a cesarean section involves the incision and ligation of uterine feeding vessels, potentially resulting in devascularization, vascular fragility, and scarring or fibrosis of uterine tissue. Consequently, these processes may lead to increased vascular resistance and reduced uterine blood flow [11,13,14,15,17]. The increase in uterine artery resistance likely plays a subclinical role in elevating the risk of adverse pregnancy outcomes, such as preeclampsia, fetal growth restriction, low birth weight, or preterm birth in subsequent pregnancies, as demonstrated in several previous studies [3,4,5,7,8,9,13], though the increase in adverse outcomes could not be demonstrated in this study, likely due to insufficient power to address this secondary objective.

In contrast to most previous studies [15,16,17,23,24], which did not perform multivariable analyses to adjust for potential confounding factors affecting uterine artery Doppler parameters, our study employed both univariable and multivariable analyses, thereby enhancing the robustness and reliability of the findings. Among the previously published studies, only the study by Hashemi et al. [25] conducted multivariable analysis, and their findings were consistent with ours, demonstrating that previous CS was significantly associated with increased uterine artery PI in both univariable and multivariable analyses. However, their analysis did not adjust for several potentially important confounders, including placental location, ASA prophylaxis, previous abortion, and uterine curettage, all of which were accounted for in our study. Furthermore, they did not evaluate the association between previous CS and uterine artery RI or abnormal uterine artery Doppler findings as categorical variables, as performed in the present study. Collectively, despite heterogeneity among studies with respect to analytical approaches [27], the currently available evidence, together with our findings, suggests that previous CS is likely associated with increased uterine artery resistance.

Note that this study was designed as explanatory research, specifically focused on the association of cesarean section on subsequent uterine vessel health, and not aimed at identifying the association of other factors such as maternal age, gestational age, aspirin prophylaxis, etc., with uterine artery Doppler results. Therefore, the lack of significance found between uterine artery Doppler and these factors does not mean there is no true association. This is because we aimed to reduce confounders, including known factors that influence uterine artery Doppler. For example, gestational age did not appear to affect uterine artery Doppler in this study because we pre-defined a narrow gestational age range, limited to between 18 and 22 weeks. All potential confounders for uterine artery Doppler were included in the full model, even if they were not significant in the univariable analysis. Thus, caution must be exercised when interpreting our results, as the lack of significant findings for factors like maternal age, parity, or placental location does not necessarily mean there is no true association.

The potential risk factors included in the analysis to control for confounding are those previously reported to possibly influence uterine artery resistance, although many studies have shown inconclusive or conflicting results. These factors include maternal age, particularly over 35 years [36,37], parity [17,38], and aspirin prophylaxis, which may reduce uterine artery PI [39] and is recommended for the prevention of preeclampsia in at-risk women [40]. Placental location might also affect uterine artery Doppler results, as uteroplacental blood flow is expected to be poorer with an anterior placenta, particularly beneath incision scars. An anterior placental location may theoretically carry a higher risk of abnormal uterine artery Doppler study compared to other locations. Therefore, placental location should be considered as a potential risk factor. Previous abortion and curettage were also considered potential risks due to their possible risk of uterine damage. These factors were adjusted for in the analysis to determine whether a previous cesarean section is an independent factor affecting uterine artery Doppler. Thus, the significance of their effects on uterine artery Doppler should not be interpreted solely based on our results, as they were not systematically included as comparison parameters.

**The strength** of this study comes from the high reliability of its conclusions. This reliability is supported by several factors, including an adequate sample size for the primary objective, there is strong consistency across the three measurement endpoints, the study follows a prospective design, and there is rigorous control of confounding variables. Moreover, this research is groundbreaking as it is the first to compare Doppler indices of fetal circulation in the umbilical artery and vein between pregnancies following a cesarean section and a control group.

**The limitations** are as follows: Although the sample size was adequate for the primary objective, it may have been insufficient to detect a significant effect of previous cesarean section on pregnancy outcomes. In addition, residual confounding may still have existed, particularly due to differences in baseline characteristics that could not be fully adjusted for in the multivariable analysis. Finally, the lack of assessment of inter- and intra-observer variability in uterine artery Doppler measurements may limit the generalizability of the findings. Nevertheless, all measurements were performed by the authors, who are specialists in maternal-fetal medicine, in accordance with the standardized guidelines recommended by the SMFM.

**Clinical implication**: From a clinical perspective, this study is not yet likely to be clinically helpful, as it is an explanatory etiologically study aimed at identifying the in-depth effects of cesarean section on subclinical changes. Nevertheless, it provides evidence to support further research, particularly in the use of uterine artery Doppler to differentiate patients with a previous cesarean section and triage them as being at low or high risk for poor obstetric outcomes. Additionally, our results may help clinicians exercise greater caution when considering cesarean section in the absence of clear indications. Clinically, if further studies confirm these findings or if they are reproducible through external validation, the assessment of Uterine artery Doppler could be an attractive and helpful tool for managing pregnant women with a previous cesarean section. This is because it is non-invasive and, importantly, can be performed during routine anomaly screening at mid-pregnancy without additional effort or cost. However, whether this statistical significance translates into clinical significance among pregnant women with a history of cesarean section remains to be clarified.

**Research implication**: According to our findings, cesarean section may have a subclinical detrimental effect on uterine artery health, as indicated by an increase in uterine artery resistance. This could theoretically place women at a higher risk of abnormal placentation in subsequent pregnancies. Future research should focus on whether this insight could be helpful in categorizing the risk of conditions such as placenta accreta syndrome or placental insufficiency.

## 5. Conclusions

Pregnant women with a previous cesarean section show significantly increased uterine artery resistance, as indicated by higher Uterine artery PI, Uterine artery RI, and a higher rate of women with Uterine artery PI above the 95th percentile in the mid-trimester, suggesting that cesarean section may negatively impact uterine artery health. However, no significant adverse clinical outcomes were observed. This may be attributable to the limited sample size for assessing clinical outcomes, or alternatively, the effect may be modest and no clinical significance.

## Figures and Tables

**Table 1:** Descriptive baseline characteristics of the study population.

Baseline Characteristic	Unscarred Uterus (N = 277)	Previous CS (N = 74)	*p*-Value
Age (years); mean ± SD	30.64 ± 0.30	33.6 ± 0.60	<0.001
Parity			<0.001
0	156 (56.31%)	0	
1	85 (30.69%)	45 (60.81%)	
≥2	36 (13.00%)	29 (39.19%)	
Prepregnant BMI (kg/m^2^); mean ± SD	22.78 ± 0.27	24.22 ± 0.51	0.016
Number of previous vaginal births			<0.001
0	181 (65.34%)	69 (93.24%)	
1	77 (27.80%)	2 (2.71%)	
≥2	19 (6.86%)	3 (4.05%)	
Number of previous cesarean section			<0.001
0	277 (100%)	0 (0%)	
1	0	65 (87.84%)	
2	0	9 (12.16%)	
Women with previous abortion			0.028
0	235 (84.84%)	53 (71.62%)	
1	34 (12.27%)	16 (21.62%)	
2	8 (2.89%)	5 (6.75%)	
Women with previous uterine curettage			0.030
0	255 (92.06%)	62 (83.78%)	
1	19 (6.86%)	12 (16.22%)	
2	3 (1.08%)	0 (0%)	
Gestational age at enrollment (week); mean ± SD	20.59 ± 0.05	20.54 ± 0.10	0.632
Gestational age at delivery (week); mean ± SD	38.78 ± 0.11	38.29 ± 0.10	0.020
Placental location			0.071
Anterior	98 (35.38%)	37 (50.00%)	
Posterior	132 (47.65%)	31 (41.89%)	
Fundal	24 (8.67%)	4 (5.41%)	
Lateral	23 (8.30%)	2 (2.70%)	
Aspirin prophylaxis	38 (13.71%)	2 (2.70%)	<0.001

**Table 2:** Placental circulation Doppler indices.

Doppler Parameters	Unscarred Uterus (N = 277)	Previous CS (N = 74)	*p*-Value
***Uterine artery indices***			
Uterine artery PI (MoM); mean ± SD	0.95 ± 0.02	1.11 ± 0.04	<0.001
Uterine artery PI > P95; number (%)	17 (6.14)	10 (13.51)	0.034
Uterine artery RI (MoM); mean ± SD	0.56 ± 0.01	0.62 ± 0.01	<0.001
Bilateral uterine artery notching [Number; %]	4; 1.44	3; 4.05	0.165
***Umbilical artery indices***			
Umbilical artery PI (MoM); mean ± SD	1.21 ± 0.01	1.18 ± 0.02	0.190
Umbilical artery RI (MoM); mean ± SD	0.71± 0.00	0.70 ± 0.01	0.381
Umbilical artery systolic/diastolic (S/D) ratio; mean ± SD	3.66 ± 0.05	3.54 ± 0.09	0.270
***Umbilical vein indices***			
Umbilical vein diameter (cm); mean ± SD	0.30 ± 0.00	0.29 ± 0.00	0.087
Umbilical vein maximum velocity cm/s; mean ± SD	20.35 ± 0.27	21.17 ± 0.60	0.180
Umbilical vein blood flow mL/min; mean ± SD	43.96 ± 0.87	43.25 ± 1.57	0.702

**Table 3:** Univariate and multivariate regression analysis of uterine artery indices.

	Univariable			Multivariable		
**Mean uterine artery PI**	**Coef.**	**95% CI**	***p*** **-value**	**Coef.**	**95% CI**	***p*** **-value**
Previous cesarean section	0.158	0.083 to 0.232	<0.001	0.190	0.104 to 0.277	<0.001
Maternal age (year)	0.002	−0.004 to 0.008	0.489	0.000	−0.007 to 0.007	0.990
Gestational age of scan (week)	0.012	−0.025 to 0.050	0.522	0.011	−0.025 to 0.048	0.542
Pre-pregnancy BMI (kg/m^2^)	−0.005	−0.012 to 0.002	0.126	−0.007	−0.014 to 0.000	0.057
Previous abortion	0.046	−0.035 to 0.128	0.262	−0.007	−0.126 to 0.111	0.903
Previous curettage	0.104	−0.001 to 0.209	0.052	0.107	−0.038 to 0.252	0.149
Acetylsalicylic acid (ASA) prophylaxis	−0.039	−0.137 to 0.059	0.438	−0.008	−0.128 to 0.113	0.899
Parity (reference: 0)						
▪ 1	0.006	−0.064 to 0.075	0.876	−0.062	−0.146 to 0.021	0.144
▪ 2	0.062	−0.024 to 0.148	0.157	−0.065	−0.190 to 0.060	0.309
Placenta location (reference: posterior)						
▪ Anterior	−0.034	−0.101 to 0.034	0.328	−0.044	−0.111 to 0.022	0.192
▪ Fundus	−0.099	−0.218 to 0.019	0.100	−0.101	−0.218 to 0.016	0.091
▪ Lateral	−0.161	−0.286 to −0.037	0.011	−0.130	−0.254 to −0.006	0.041
**Mean uterine artery RI**	**Coef.**	**95%CI**	***p*** **-value**	**Coef.**	**95%CI**	***p*** **-value**
Previous cesarean section	0.052	0.030 to 0.074	<0.001	0.058	0.033 to 0.084	<0.001
Maternal age (year)	0.001	−0.0003 to 0.003	0.108	0.001	−0.001 to 0.003	0.511
Gestational age of scan (week)	0.001	−0.010 to 0.012	0.830	0.001	−0.010 to 0.012	0.873
Pre-pregnancy BMI (kg/m^2^)	−0.002	−0.004 to 0.000	0.101	−0.002	−0.004 to 0.000	0.026
Previous abortion	0.016	−0.008 to 0.040	0.195	0.007	−0.028 to 0.042	0.713
Previous curettage	0.026	−0.005 to 0.057	0.099	0.020	−0.022 to 0.063	0.351
ASA prophylaxis	−0.008	−0.037 to 0.021	0.603	0.001	−0.035 to 0.036	0.964
Parity (reference: 0)						
▪ 1	0.010	−0.011 to 0.030	0.353	−0.013	−0.038 to 0.011	0.291
▪ 2	0.020	−0.006 to 0.045	0.130	−0.022	−0.059 to 0.015	0.240
Placenta location (reference: posterior)						
▪ Anterior	−0.009	−0.029 to 0.011	0.378	−0.012	−0.031 to 0.008	0.241
▪ Fundus	−0.034	−0.069 to 0.001	0.060	−0.032	−0.066 to 0.002	0.068
▪ Lateral	−0.054	−0.091 to −0.017	0.004	−0.044	−0.081 to −0.008	0.017
**Uterine artery PI > 95th centile**	**OR**	**95%CI**	***p*** **-value**	**OR**	**95%CI**	***p*** **-value**
Previous cesarean section	2.390	1.045 to 5.467	0.039	3.517	1.139 to 10.859	0.029
Maternal age (year)	1.000	0.927 to 1.079	0.990	0.998	0.907 to 1.099	0.975
Gestational age of scan (week)	0.947	0.592 to 1.515	0.820	0.906	0.548 to 1.499	0.702
Pre-pregnancy BMI (kg/m^2^)	0.949	0.862 to 1.045	0.288	0.946	0.847 to 1.057	0.328
Previous abortion	1.338	0.517 to 3.465	0.548	0.400	0.053 to 3.049	0.377
Previous curettage	2.312	0.814 to 6.562	0.115	3.024	0.399 to 22.933	0.284
ASA prophylaxis	0.281	0.037 to 2.130	0.219	0.285	0.030 to 2.686	0.273
Parity (reference: 0)						
▪ 1	0.693	0.265 to 1.816	0.456	0.358	0.104 to 1.238	0.105
▪ 2	1.696	0.659 to 4.366	0.274	0.798	0.147 to 4.336	0.794
Placenta location (reference: posterior)						
▪ Anterior	0.541	0.226 to 1.296	0.168	0.493	0.202 to 1.206	0.121
▪ Fundus	0.661	0.144 to 3.031	0.594	0.620	0.130 to 2.946	0.547
▪ Lateral	1.000	-	-	1.000	-	-

**Table 4:** Pregnancy outcomes.

Outcome	Unscarred Uterus (N = 277)	Previous CS (N = 74)	*p*-Value
Birthweight, grams; mean ± SD	3092.19 ± 27.69	3101.86 ± 52.53	0.873
Placental weight, grams; mean ± SD	646.03 ± 8.42	650.86 ± 17.32	0.080
Preeclampsia; number (%)	8 (2.89%)	2 (2.70%)	0.932
Fetal growth restriction; number (%)	22 (7.94%)	6 (8.11%)	0.963
Low birth weight; number (%)	25 (9.03%)	6 (8.11%)	0.805
Preterm birth; number (%)	19 (6.86%)	6 (8.11%)	0.711
Oligohydramnios; number (%)	2 (0.72%)	1 (1.35%)	0.601
Total adverse antenatal outcomes; number (%)	46 (16.61%)	13 (17.57%)	0.844

## Data Availability

The datasets analyzed during the current study are available from the corresponding author upon reasonable request.

## Abbreviations

The following abbreviations are used in this manuscript:

ACOG	American College of Obstetricians and Gynecologists
ASA	Acetylsalicylic acid
BMI	Body mass index
CS	Cesarean section
CI	Confidence interval
ISUOG	International Society of Ultrasound in Obstetrics and Gynecology
OR	Odds ratio
PI	Pulsatility index
PSV	Peak-systolic velocity
RI	Resistance index
SMFM	Society for Maternal-Fetal Medicine
UVF	Velocity of umbilical vein flow
